# Contrast-Enhanced Ultrasound in the Diagnosis of Hepatocellular Carcinoma and Intrahepatic Cholangiocarcinoma: Controversy over the ASSLD Guideline

**DOI:** 10.1155/2015/349172

**Published:** 2015-05-18

**Authors:** Le-Hang Guo, Hui-Xiong Xu

**Affiliations:** Department of Medical Ultrasound, Shanghai Tenth People's Hospital, Tongji University School of Medicine, Shanghai 200072, China

## Abstract

Hepatocellular carcinoma (HCC) and intrahepatic cholangiocarcinoma (ICC) are both regarded as primary liver cancers, having different biological behaviors and prognoses. Correct differentiation between them is essential for surgical planning and prognosis assessment. In 2005, the American Association for the Study of Liver Diseases (AASLD) recommended that noninvasive diagnosis of HCC is achievable by a single dynamic technique (including contrast-enhanced ultrasound (CEUS)) showing intense arterial uptake followed by washout of contrast in the venous-delayed phases. However, CEUS has been dropped from the diagnostic techniques in the latest AASLD guideline according to the opinion of some authors from Europe that CEUS may offer false positive HCC diagnosis in patients with ICC. Since the update of AASLD guideline has been released, increased attention has been paid to this interesting topic. Remarkable controversy over this issue is present and this removal was not well received in Europe and Asia. This commentary summarized the opinions for the role of CUES in differentiation between HCC and ICC in recent years. It is concluded that prospective studies with strict design and large case series are mandatory to solve the controversies and stratification of ICC in terms of tumor size and liver background is also essential.

## 1. Background

Hepatocellular carcinoma (HCC) is the sixth most common neoplasm worldwide. Its incidence has increased in recent years and it is currently the leading cause of death in patients with cirrhosis. Intrahepatic cholangiocarcinoma (ICC) is a rare liver tumor, and its incidence rate has also increased in recent years. Although both ICC and HCC are regarded as primary liver cancers, they have different biological behaviors and prognoses. Correct differentiation between them is essential for surgical planning and prognosis assessment [1–3]. In the recent decade, contrast-enhanced ultrasound (CEUS) has been introduced into clinical practice for diagnosis of focal liver lesion (FLL). Numerous prospective studies including several multicenter studies with large case series have already confirmed the value of CEUS in diagnosis of FLL, and it is widely accepted that CEUS is at least comparable with contrast-enhanced computed tomography (CECT) and contrast-enhanced magnetic resonance imaging (CEMRI) in characterization and detection of FLLs [4–7].

## 2. CEUS for ICC

### 2.1. Ultrasound and CEUS

Ultrasound (US) has been regarded as the first line imaging modality for detection of FLL, whereas it is difficult for baseline US to distinguish between them because ICC has no specific features [2, 3, 8–10]. CEUS, with the use of a low acoustic power contrast-specific mode and a gas-filled microbubble contrast agent, allows visualization of macro- and microcirculation of a targeted organ or lesion continuously. The CEUS vascular phases are usually classified into arterial (8–30 s from contrast agent injection), portal (31–120 s), and late (121–360 s) phases [2–5, 10, 11]. The CEUS enhancement patterns of 50 mass-forming ICCs were retrospectively analyzed by Chen et al. [2] and were compared with 50 HCCs. In their study, ICC typically produced a peripheral rim-like enhancement in the arterial phase of CEUS, which accounted for 50% of ICCs. The other enhancement patterns were heterogeneous hypoenhancement (24%), heterogeneous hyperenhancement (20%), and homogeneous hyperenhancement (6%). However, for HCC, heterogeneous (58%) and homogeneous (38%) hyperenhancement were the most common findings in the arterial phase; rim-like hyperenhancement only accounted for 4% of HCCs. In the late phase, all 50 ICCs and 50 HCCs were hypoenhanced. Time-intensity curve (TIC) demonstrated that the intensities of the peripheral and central portions of the ICCs were lower than those of HCCs. In addition, ICCs showed washout more quickly than HCCs in portal and late phases. The diagnostic performance of both readers in terms of the area under the receiver operating characteristic curve (AUROC) (0.745 versus 0.933 for reader 1, and 0.803 versus 0.911 for reader 2), sensitivity (28% versus 90%, and 44% versus 82%), and accuracy (64% versus 90%, and 71% versus 90%) improved significantly after CEUS (all *P* < 0.05). The interobserver agreement increased from kappa value (*κ*) = 0.575 at BUS to *κ* = 0.720 after CEUS. Therefore, CEUS increases the diagnostic performance in differentiation between ICC and HCC significantly, in comparison with conventional ultrasound.

Similar results have been found in the related studies and the typical CEUS manifestations for HCC and ICC are summarized as follows [3–10].

#### 2.1.1. HCC

- Hyperenhancement in the arterial phase is usually homogeneous and intense but may be inhomogeneous in larger nodules (>5 cm).
- Washout in late phase is observed in about half of HCC, but more rarely in small nodules (20–30% in those 1-2 cm, 40–60% in those 2-3 cm).
- The median time of onset of washout was reported to be 2 min and it is longer in well differentiated HCC than in poor differentiated HCC.

#### 2.1.2. ICC

- ICCs have a variety of patterns in the arterial phase with the majority showing peripheral rim-like hyperenhancement, while all show washout in the late phase, in contrast to the late enhancement on CECT or CEMRI.
- Early washout at CEUS (usually before 60 s).

Intraductal-growing ICC is another type of ICC, which often shows homogeneous hyperenhancement in the arterial phase and washout in the late phase. This type is easy to be differentiated from HCC since it is always located in dilated bile duct whereas HCC is seldom associated with bile duct dilation. Periductal infiltration type of ICC is hard to be detected before surgery; it is also a rare type ICC [10].

### 2.2. Multimodality Comparison

The vascular structures in the solid tumor are always disorganized and leaky [12–14]. The vascular permeability in turn drives tumor-induced angiogenesis and blood flow disturbances, which also facilitates the CT and MRI contrast agents diffusing over time from hyperpermeable intratumoral blood vessels to the interstitium of the tumor, whereas the ultrasound contrast agents are blood pool agents with large size and thus remain in the blood vessels [15, 16]. Direct comparison between them in the vascular phase is possible, whereas the comparison in the late phase is difficult. Persistent enhancement in the late phase is a typical pattern for ICC on CT and MRI, whereas nearly 100% ICCs show washout in late phase on CEUS [11, 17]. Chen et al. [11] carried out a comparison study between CEUS and CECT in diagnosis of ICC. The enhancement patterns of ICC on CEUS are consistent with those on CECT in the arterial phase, indicating that it is pathology, not the adopted imaging technology, which determines the enhancement pattern. In the portal phase, ICC fades out more obviously on CEUS than on CECT. In the late phase, all ICCs show washout on CEUS whereas sustained enhancement on CECT. CEUS (accuracy, 80%) has the same accuracy as CECT (accuracy, 67.5%) in diagnosing ICCs and can be used as a new modality for the characterization of ICC.

### 2.3. ICC: Pathological Correlation with CEUS Pattern

As mentioned above, ICC has different pathological subtypes and the CEUS enhancement pattern is varying. It was confirmed that CT and/or MRI findings of ICC were correlated with pathological components. The hyperenhancing areas always indicated a large number of tumor cells and the delayed enhancement corresponded to fibrotic stroma at pathological examination. Xu et al. [10] found that the hyperenhancing areas on CEUS corresponded to more tumour cells for mass-forming ICCs. There are scarce tumour cells in the centre portion of the tumour and fibrosis is prominent. Microscopically, scarce tumour cells are present in both the peripheral portion and the centre portion. In periductal-infiltrating ICC, fibrosis was more commonly found, whereas in intraductal-growing ICCs, abundant tumor cells were found. Therefore, the imaging findings of ICC on CEUS were related to the degree of carcinoma cell proliferation at pathology; hyperenhancing areas in ICC always indicate increased density of tumor cells.

## 3. AASLD Guideline Opinion

In 2005, the American Association for the Study of Liver Diseases (AASLD) endorsed that noninvasive diagnosis of HCC is achievable by a single dynamic technique (including CEUS) showing intense arterial uptake followed by washout of contrast in the venous-delayed phases [1]. Afterwards, CEUS has also been introduced into other important guidelines and recommendations for the diagnostic work-up of HCC in patients with cirrhosis: the Asian Pacific Association for the Study of the Liver (APASL) consensus recommendations on HCC [18]; the recommendations of the Japanese Society of Hepatology [19]; the European Federation of Societies for Ultrasound in Medicine and Biology (EFSUMB) guidelines 2008 [6]; and the World Federation for Ultrasound in Medicine and Biology- (WFUMB-) EFSUMB guidelines 2012 [4, 5]. Regarding the contrast agent, the APASL guidelines accept the use of Levovist (Schering, Berlin, Germany) or Sonazoid (GE Healthcare, Milwaukee, WI, USA) as contrast agents, the Japanese guidelines accept only the use of Sonazoid, and the WFUMB-EFSUMB guidelines accept both SonoVue (Bracco, Milan, Italy) and Sonazoid as contrast agents. The APASL guidelines recommend the use of either CT or MRI as a first-line technique and resort to CEUS for second/third line in the absence of typical diagnostic CT and/or MRI patterns, whereas the JSH guidelines accept CEUS even as a first-line option.

However, CEUS has been dropped from the diagnostic techniques in the latest AASLD guideline according to the opinion of some authors from Europe that CEUS may offer false positive HCC diagnosis in patients with ICC. Another reason is that liver CEUS is not approved by Food and Drug Administration (FDA) in the USA. The latter factor indicates that the published results might not be applicable to a North American population. Therefore, the new AASDL guideline has negative opinion on the use of CEUS to make differentiation between HCC and ICC [20].

## 4. Current Controversy over AASLD Guideline

Since the update of AASLD guideline has been released, increased attention has been paid to this interesting topic. Remarkable controversy over this issue is present and this removal was not well received in Europe and Asia. It is questioning whether CEUS should be entirely removed from the important guidelines based on a single, relatively small-scale study that is the source of negative opinion from AASLD [21–28]. Currently, the opinion of negative side was based on limited cases of ICC, and even the limited cases were scattered in different centers, whereas the opinion of positive side was based on the widest ICC/HCC comparison case series. However, subanalysis was absent in both sides.

### 4.1. Negative Opinion

In 2010, Vilana et al. [21], on behalf of the Barcelona Clinic Liver Cancer (BCLC) group, analyzed the CEUS enhancement patterns of 21 patients with ICCs from 2003 to 2009 and then concluded that CEUS was not able to be used to differentiate ICC and HCC since the enhancement patterns were similar with HCC in 10 cases that they showed homogeneous arterial hyperenhancement followed by washout. They also concluded that CEUS should not be used as the sole imaging technique for conclusive HCC diagnosis. The potential risk of positive diagnosis of HCC in cirrhosis by CEUS should therefore be kept in high consideration.

In 2012, Bohle et al. [23] retrospectively analyzed the CEUS pattern of 39 patients with HCC, 11 patients with ICCs, 3 patients with Klatskin tumors, and 4 patients with gallbladder carcinomas; they found that HCC and ICC differ to some extent in their CEUS enhancement pattern. Incomplete arterial hyperenhancement is more often seen in ICC. A rim sign seems to be specific for ICC but is only rarely present. Due to overlapping characteristics, a reliable differentiation between the two tumor types by CEUS alone is very often not possible.

### 4.2. Equivocal Opinion

In 2013, Galassi et al. [24] analyzed the CEUS patterns of 25 small ICCs from three Italian centers between 2003 and 2011, which means less than one case in each center per year. They found that CEUS was at risk of misdiagnosis of ICC for HCC in a significantly higher number of cases than in CT (52% versus 4.2%) and MRI (52% versus 9.1%). In the arterial phase, ICC lacked global hyperenhancement in approximately 50% of cases at CEUS and the degree of intensity of washout in the late phase was marked in 24% of nodules. They concluded that CEUS misdiagnosed as HCC a significantly higher number of ICC lesions in cirrhotic patients than CT and MRI. However, some CEUS contrast features can help suspect ICC, especially in some cases with inconclusive CT or MRI.

### 4.3. Positive Opinion

In 2012, Barreiros et al. [25] firstly commented on the AASLD guideline regarding removal of CEUS for diagnosis of HCC. In the opinion of the authors, CEUS should be part not only of most, but all international HCC guidelines. Giorgio et al. [26] made a comment on the study of Galassi et al. [24] and concluded it was an exaggerated fear for misdiagnosis of ICC in cirrhosis at CEUS. They endorsed the study of Chen et al. [2] that it was still the widest ICC/HCC comparison case series, even if not specifically devoted to ICC on cirrhosis, CEUS significantly improved the diagnostic performance in the differentiation between ICC and HCC.

In 2013, Korean Association for the Study of CEUS also commented on this issue. They regarded this removal as controversial and not well received in Europe and Asia [27]. Bohle et al. [23] also commented on this issue and concluded that there was no need to remove CEUS from the AASLD practice guidelines as part of the diagnostic algorithm for HCC in cirrhotic patients. Limited market availability (i.e., liver CEUS is not approved by FDA in the USA) is also not a justification to eliminate a valuable diagnostic tool from the algorithm. CEUS should not be considered as a “second-line” imaging technique in the patient care.

Li et al. [29] firstly evaluated the usefulness of CEUS in differentiating ICC from HCC in cirrhotic patients. They found that analysis of detailed temporal enhancement features on CEUS is helpful to differentiate ICC from HCC in cirrhosis. If a nodule in cirrhotic liver displays hyperenhancement in the arterial phase followed by early and marked washout in the portal phase, the nodule is highly suspicious of ICC rather than HCC since, in comparison with ICC, the washout in portal phase is moderate and sometimes even shows iso-enhancing for HCC. Their results provided the latest evidence to rebuke the opinion from AASLD.

## 5. Discussion and Conclusion

The controversy over AASLD guideline regarding the differentiation between HCC and ICC using CEUS continues. When reanalyzing the data of Vilana et al. [21], we found that 52.4% of their cases showed periphery hyperenhancement in arterial phase, 71.4% showed hypoenhancement in portal phase, and 100% showed hypoenhancement in late phase. All of the features are characteristic for ICC instead of HCC. In addition, no HCC was included and no comparison study was performed; thus no data supported the conclusion directly. The characteristic pattern of periphery hyperenhancement for ICC in arterial phase was overlooked, as well as quick washout in portal and late phases. The low incidence of ICC was also overlooked; in an analysis of 993 adult cirrhotic liver explants with liver cancer, ICC was only found in 1% of all the cases [22]. The study of BCLC group was carried out in a long period and only 3 cases per year were evaluated, which aroused doubt about their experience and also indicated that the risk for misdiagnosis is only less than 3/year [21]. The low risk for misdiagnosis could also happen for CECT or CEMRI whereas they were not removed from the diagnostic work-up. Additionally, the CEUS pattern for ICC would suggest a diagnosis of malignancy with almost 100% specificity since nearly 100% showed washout in late phase, whereas the pattern of CT or MRI might not be specific for malignancy (washin followed by sustained enhancement) that some had been misdiagnosed as hemangioma or abscess. This is an important point that biopsy might not be feasible in all the patients. In addition, it should be pointed out that the study was not a prospective and controlled study design. Finally, to overcome the uncertainty in relation to the complex visual investigation of dynamic loops at CEUS, quantification of CEUS is necessary, which may facilitate differential diagnosis between ICC and HCC [30, 31]. Regarding the study of Bohle et al. [23], the conclusion was obviously paradox and was not based on the results. In addition, no diagnostic test was performed to differentiate HCC and ICC.

Barreiros et al. [25] refuted the opinion of AASLD with the following points. Firstly, CEUS has been introduced more than 10 years for liver imaging in many European and Asian countries, but FDA approval in the US is still lacking. Thus the evidence for the AASLD opinion is scarce. Secondly, ICC is a rare tumour in liver cirrhosis (about 1–3% of newly developed tumors); thus it should not overestimate the risk for misdiagnosis. Thirdly, when referring the study of Vilana et al. [21], it was a nonprospective and noncontrolled study design. Therefore, the quality of the study and the clinical consequences of this possible risk do not seem to justify the complete removal of CEUS from the imaging armamentarium. In addition, the study was carried out without enough experience; the investigators were not well trained, and CEUS was not performed in expert hand.

Therefore, the evidence for positive opinion is accumulating. Prospective studies with strict design are mandatory to solve the controversies. Future studies should focus on the imaging features of ICC in healthy livers and cirrhotic livers. Prospective case control studies with large case series are needed. Stratification of ICC in terms of tumor size and liver background is also essential. Finally, CEUS has the reason to retain in the guidelines because there is no radiation exposure to the patient, negligible nephrotoxicity, and a low risk of allergic and hypersensitivity reactions. CEUS is also applicable when CT or MRI is contraindicated.
